# Kinetics of Silver Accumulation in Tissues of Laboratory Mice after Long-Term Oral Administration of Silver Nanoparticles

**DOI:** 10.3390/nano11123204

**Published:** 2021-11-26

**Authors:** Anna A. Antsiferova, Marina Yu. Kopaeva, Vyacheslav N. Kochkin, Pavel K. Kashkarov

**Affiliations:** 1 National Research Center “Kurchatov Institute”, 1, Akademika Kurchatova sq., 123182 Moscow, Russia; m.kopaeva@mail.ru (M.Y.K.); Kochkin_VN@nrcki.ru (V.N.K.); kashkarov_pk@nrcki.ru (P.K.K.); 2 Moscow Institute of Physics and Technologies, 9, Institutskii Lane, 141700 Dolgoprudny, Moscow Region, Russia; 3 Department of Physics, Lomonosov Moscow State University, GSP-1, Leninskiye Gory, 119991 Moscow, Russia

**Keywords:** silver, silver nanoparticles, accumulation, mammals, testis, brain, lungs, neutron activation analysis, blood–testicular barrier

## Abstract

Since ancient times, silver has been known for its pronounced bactericidal, antiviral and fungicidal properties. Currently, nanoparticles of this metal are widely used in the food, light and pharmaceutical industries, as well as in medicine. Silver in any form can have a toxic effect not only on pathogens, but also on healthy cells. The biological activity and bioavailability of silver preparations depend on the degree of their solubility in water. In addition, the maximum permissible concentration of soluble forms of silver is an order of magnitude lower than that of insoluble forms. This makes nanoparticles of silver with a hydrophilic coating that form stable colloidal solutions in aqueous media potentially unsafe objects. In this work, we studied the kinetics of the accumulation of silver nanoparticles with an average size of 34 ± 5 nm stabilized with polyvinylpyrrolidone in the organs of laboratory C57Bl/6 mice. The administration of nanoparticles was carried out orally for 30, 60, 120 and 180 days at the dose of 50 µg/day/animal. All the mice developed and gained weight normally during the experiment. No adverse effects were observed. Determination of the silver content in biological tissues of mammals was accomplished by neutron activation analysis. The masses and concentrations of silver in the brain and its different sections (hippocampus, cerebellum, cortex and remnants), as well as in the lungs, testes, liver, blood, kidneys, spleen and heart, were determined. The injection times at which the accumulation curves reached saturation were established. An extremely high accumulation of silver in the testes was shown at 120 days of administration, and a significant accumulation of silver in the lungs and brain was observed. The accumulation of silver in all parts of the brain except the cortex was significant, and its trend was similar to that in the whole brain.

## 1. Introduction

Since ancient times, silver has been known for its antiseptic properties. It was used for medicinal purposes in ancient Egypt and Mesopotamia. Moreover, Hindu Ayurvedic texts mention the disinfection of water by immersing hot silver into it or by prolonged contact with metallic silver under normal conditions [1]. Until 1800, the use of exclusively metallic silver was documented. However, with the turn of the 19th century, its salts and colloidal solutions, such as argyrol and protargol, began to be used. Since the beginning of the 2000 s, in relation to the development of nanotechnology, silver nanoparticles began to be actively used in food, pharmaceutical and light industries, as well as in medicine [2].

In general, silver in all forms exhibits antibacterial, antiviral and fungicidal properties. However, the toxic properties of silver and, in particular, its nanoparticles, can affect not only pathogens, but also healthy cells [3,4]. It is now clear that the biological activity of silver drugs depends on the degree of their solubility, since in organisms, they interact with the aqueous phase. Previous studies have proposed to distinguish the maximum permissible doses of silver content in water, taking into account the hydrophilicity of the corresponding compounds. The maximum allowable concentration for metallic silver was determined to be 0.1 mg/m^3^, while for its soluble forms, it is 0.01 mg/m^3^ [5]. Thus, the transition to a soluble form, for example, to colloidal solutions, increases the bioavailability of silver and also enhances its toxicity. Therefore, special attention should be paid to stable solutions of silver nanoparticles coated with a hydrophilic stabilizer. The toxicity of silver is associated with the interaction between its ions and negatively charged groups of cellular biomacromolecules [6]. Moreover, nanoparticles can increase the permeability of the cell membrane and disrupt the membrane integrity for both mammalian and bacterial cells [7,8,9] and act as a depot of ions [10]. Ultimately, silver nanoparticles can influence cell metabolism and lead to genetic changes and apoptosis [11,12]. It is generally accepted that silver nanoparticles induce the generation of reactive oxygen species, which cause the aforementioned unfavorable processes [13]. With prolonged exposure to silver, including its nanoforms, for example, in the treatment of trophic ulcers and other purulent skin lesions, such conditions known in clinical practice as argyria and argyrosis can appear [14], which present as discoloration of the skin and mucous membranes. Generalized argyria developed in a patient with oral ulcers who had her tongue painted with 10% silver nitrate repeatedly for 1 year (~0.2 mg/kg bw/day) [15]. In general, argyria and argyrosis are considered as cosmetic defects. Long-term exposure to silver and its nanoparticles in relatively high doses can occur in environments where silver recycling occurs. For example, it can occur in food chains when fertilizers based on nanosilver are introduced. Understanding the processes that occur during the interaction between silver in its nanoform and biological organisms is extremely important with regard to its introduction into the environment.

To assess the toxicity of silver in various forms, data on its bioaccumulation in various organs are very important. Silver accumulated in organs in ionic and nanoforms can have a toxic effect on tissues, as well as be transported to other tissues through the bloodstream. It is very important to study the bioaccumulation of silver to predict possible toxic effects on certain tissues. Moreover, it is highly important to study oral exposure to silver nanoparticles due to the modelling of oral intake of medications based on silver nanoparticles [16]. In our previous in vivo studies on mice, for the first time, an extremely low level of elimination of silver nanoparticles from the brain was found [17]. Therefore, we decided to continue this line of research and conduct a more detailed examination of various organs and their parts. In the present work, we studied the kinetics of silver accumulation in the organs of laboratory mice after long-term oral exposure to silver nanoparticles.

## 2. Materials and Methods

As a source of silver nanoparticles, we used a commercially available food supplement “Argovit S” with an initial concentration of 10 mg/mL (Vector-Vita, Novosibirsk, Russia). This is a food supplement recommended for humans to protect against gastrointestinal diseases [18]. It can also be used in veterinary and agriculture applications. The particles were stabilized with polyvinylpyrrolidone. Nanoparticle size was determined by dynamic light scattering (DLS) (Malvern Zetasizer, Malvern, UK) and transmission electron microscopy (TEM) (Thermo Fisher Scientific, Waltham, MA, USA). The stability of the preparation was also studied after storage in the dark at a temperature of +2 °C for 1 year using the DLS method.

As a mammalian model, we used male C57Bl/6 mice with an initial body weight of 20–22 g starting from the age of 2 months, and they were obtained from the “Stolbovaya” branch of the Federal Medical Biological Agency of Russia.

Pure water Osmoteck 40-3-2 (OOO “Pharmsystemy”, Besedy, Moscow, Russia) was used as drinking water and for the preparation of nanoparticle solutions.

The mass of silver accumulated in the internal organs was determined by instrumental neutron activation analysis (INAA) using an IR-8 nuclear research reactor (Moscow, Russia) with a power of 8 MeV and a gamma spectrometer (ORTEC, Oak Ridge, TN, USA).

For the preparation of reference samples, we used a solution of a standard sample of silver in the form of silver nitrate with a silver ion concentration of 1 g/L (LenReaktiv, Saint Petersburg, Russia). INAA has already been successfully used in the study of gold nanoparticle biokinetics [19], as well as in silver nanoparticle biokinetics study for prenatal [20] and oral exposure for subchronic periods of administration [17,21,22].

## 3. Scheme of the Experiment

To study the size of nanoparticles by DLS, the concentration of solutions was selected to provide reproducibility of results in 10 consecutive measurements. For this, the solution was diluted 50 times with deionized water. During TEM, the nanoparticle solution was also diluted to 1 mg/mL. Before taking measurements, a drop of the nanoparticle solution was applied to a carbon grid and then dried.

**Figure 1:**
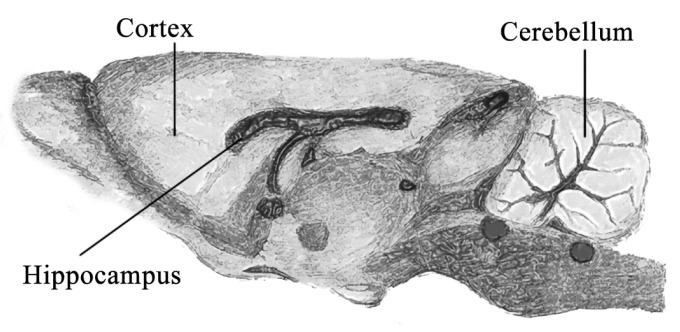
The scheme of brain indicating the studied brain departments.

All procedures with animals were conducted according to the rules of the Ministry of Health of the Russian Federation (No. 267 of 19 June 2013), and approved by the Local Ethics Committee for Biomedical Research of the National Research Center “Kurchatov Institute” (No. 01 from 10 February 2017). Throughout the experiment, the animals were kept in individual cages with unlimited access to food and water in rooms with automatically maintained temperature of 23 ± 2 °C and a 12/12-h day/night cycle. The room’s humidity was also controlled at 45 ± 10%. The changes in the body weights of the animals were monitored weekly. The mice were divided into 4 groups according to the periods of the exposure to nanoparticles: groups of 30, 60, 120, and 180 days. Each of these groups was also divided into 2 subgroups: experimental and control. The 30, 60 and 120 days of exposure groups contained 20 animals, while the 180 days group contained 24 animals. Nanoparticles were administered orally daily at 50 μg/day/animal in ad libitum drinking water. If we assume that the weight of the mouse was 25 g on average, then the animals received silver nanoparticles at a dose of 2 mg/kg bw. Drinking bowls were weighed weekly to determine the fluid intake. Thus, we estimated the amount of fluid consumed. We noticed that the animals consumed 3.8 mL of water per day. Based on this, the required amount of silver nanoparticles was dissolved in water. After the specified time of administration of nanoparticles, some of the animals were decapitated after anesthesia with isoflurane (Baxter, Puerto Rico, USA) (n = 6 (8)). The remaining animals (n = 4 in each group) were anesthetized by intramuscular injection of physiological saline (0.9% sodium chloride solution, Dalkhimpharm, Khabarovsk, Russia) containing zoletil (Virbac Sante Animale, Chambray-les-Tours, France) and rometar (Bioveta, Ivanovice na Hane, Czech Republic). Then, transcardial perfusion was performed using an Ecoline ISM1090 peristaltic pump (Ismatec, Glattbrugg, Switzerland), first with a phosphate-buffered solution (Sigma-Aldrich, St. Louis, MO, USA) with a pH of 7.4 and at room temperature, and then with 4% paraformaldehyde solution (Sigma-Aldrich, St. Louis, MO, USA) in phosphate-buffered solution. The brain, lungs, testes, liver, kidneys, blood, heart and spleen were collected from mice. Some brains were divided into the following sections: hippocampus, cerebellum, cortex and remnant (Figure 1). This protocol was based on other widely used procedures [23,24]. The cerebella were removed using a small scalpel and the remaining brain tissue was divided into the two hemispheres. These were then further dissected to obtain the cortex/hippocampus and the residual fraction. The hippocampus was identified and isolated from the cortical fraction as a complete tissue.

Tissue samples were dried in a drying cabinet for 72 h at a temperature of 75 °C for irradiation in the channel of a nuclear reactor. Then, they were placed in polyethylene containers (Eppendorf, Hamburg, Germany) at volumes of 0.2, 0.5 and 2 mL, hermetically sealed, and numbered with a moisture-resistant marker. At this time, reference samples were prepared for irradiation in the channel of a nuclear reactor. For this, cotton wool was placed in the same plastic containers (to maintain the identity of the geometry factor), and a known amount of the standard sample of silver (100 or 1000 ng per sample) was added. The containers were left open, air dried for 48 h and then hermetically sealed. Flat reference samples were also prepared. For this, a known amount of state standard sample of silver was placed on paper disks and dried in air. Next, plastic containers and reference samples were placed in aluminum cases. Each aluminum case contained one reference sample with the same geometry factor as the experimental samples, as well as 84 flat samples. Aluminum cases were suspended in a vertical channel of a nuclear reactor and irradiated for 24 h in a neutron flux of 10^12^ cm^−2^ s^−1^. After irradiation, the cases were kept in biosecurity for the decay of highly active short-lived isotopes, and then gamma-spectrometric studies of the samples were carried out for the activities of the radioactive isotope ^110m^Ag with a half-life of 250 days.

Statistical analysis was performed using GraphPad Prizm 6.01 software (La Jolla, San Diego, CA, USA). The nonparametric Kruskal–Wallis ANOVA with a post hoc Dunn’s test for multiple comparisons or Mann–Whitney U test were employed. Differences were considered significant at *p* ≤ 0.05.

## 4. Results and Discussion

**Figure 2:**
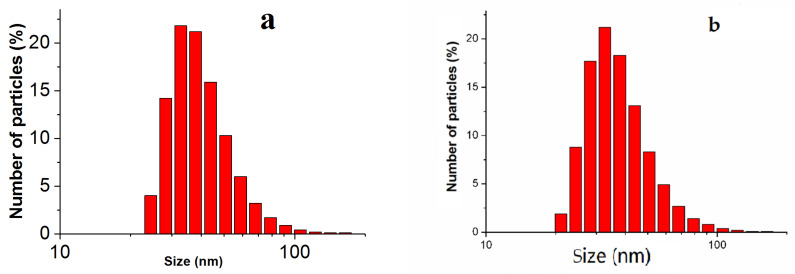
Distribution of the number of particles by size according to the DLS data: (**a**) fresh solution (the average particle size was 34 ± 5 nm); (**b**) the solution after storage for 1 year (the average particle size was 36 ± 5 nm).

**Figure 3:**
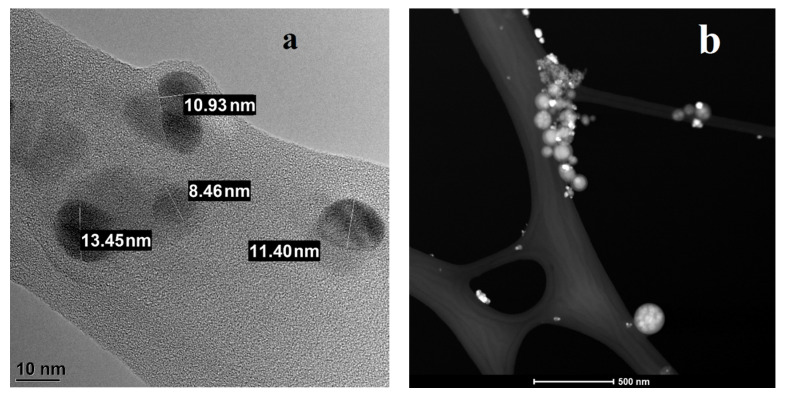
Micrograph of silver nanoparticles “Argovit S”: (**a**) in brightfield mode, scale is 10 nm, (**b**) in darkfield mode, scale is 500 nm.

According to the DLS data (Figure 2a), the average nanoparticle size was 34 ± 5 nm (polydispersity index = 0.429). The micrograph (Figure 3) shows quasi-spherical particles. Individual particles were aggregated. 

The high stability of the suspension of nanoparticles "Argovit S" was demonstrated after storage for 1 year due to the average particle size remaining practically unchanged according to DLS (Figure 2b) (polydispersity index = 0.449). The high stability of the nanoparticles is due to the polvinylpirrolidne coating that prevented particle aggregation. The coating provided solubility of the nanoparticles in the aqueous solutions due to its hydrophilicity.

During the experiment, the animals developed normally and gained weight (Figure S1, Supplementary Materials). No signs of argyria of internal organs of mice were observed after euthanasia.

Table 1 shows the absolute values of the silver content in the brain regions. The masses of the brain parts are presented in Table S1, Supplementary Materials. It can be observed that for a number of organs, the silver masses were less than 10 ng. Such measurements cannot be carried out with the use of mass spectrometry or TEM. It should be noted that INAA is a unique tool for studying the biokinetic nanoparticles of non-essential elements, as it allows one to obtain results with metrological accuracy and sensitivity up to 10^−9^ g, as well as registering activity signals from the whole organs of laboratory mammals and not just their parts, unlike most other known methods. Thus, the method is highly sensitive and representative.

**Figure 4:**
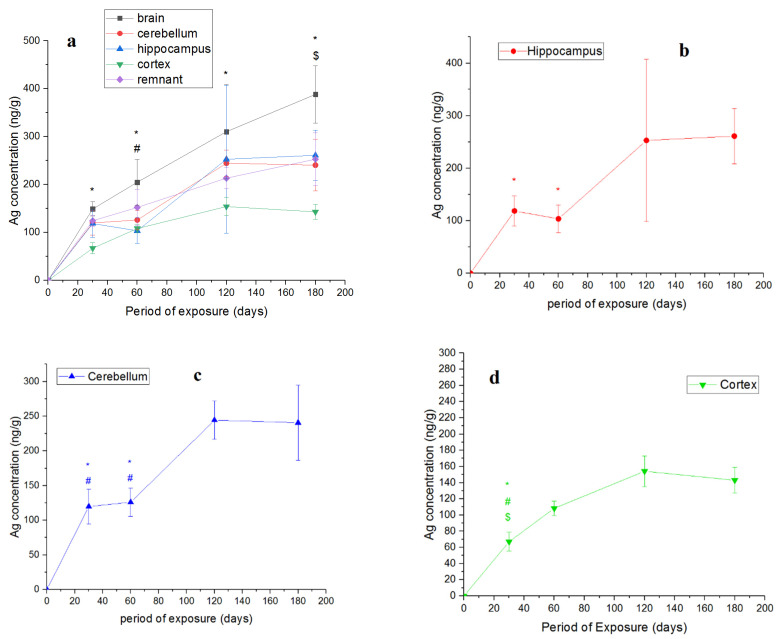

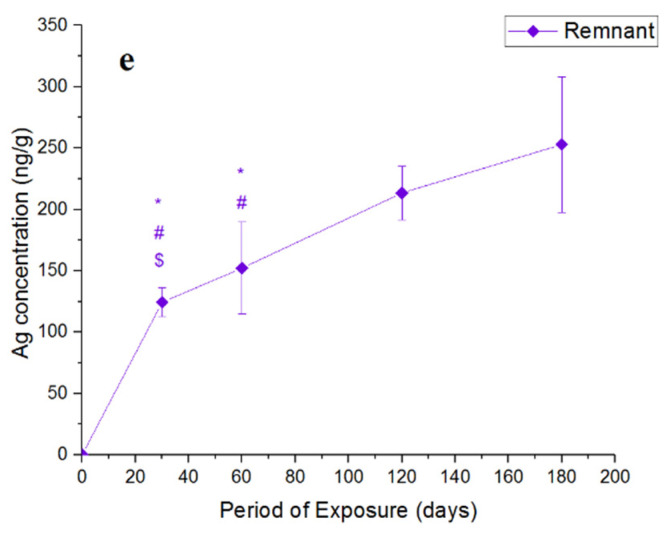
Accumulation of silver in brain and its departments: (**a**) dependence of the concentration of silver in the brain and its parts on the period of administration of silver nanoparticles (* *p* < 0.05—brain vs. cortex, ^#^ *p* < 0.05—brain vs. hippocampus, and ^$^ *p* < 0.05—cortex vs. hippocampus in each group); (**b**) hippocampus; (**c**) cerebellum; (**d**) cortex; (**e**) remnant (the points to the left denoted by * are statistically different from the 180 day point; the points to the left denoted by ^#^ are statistically different from the 120 day point; the points to the left denoted by ^$^ are statistically different from the 60 day point, * *p* < 0.05, ^#^ *p* < 0.05, ^$^ *p* < 0.05). Number of organs per time point, *n* = 4–8.

Figure 4a shows the dependence of the concentration of silver in the brain and its regions on the time of nanoparticle administration.

The silver content in the control samples was below the detection limit, so the curves were drawn from the origin.

The lowest concentration of silver was observed in the cortex. After 120 days, the concentration of silver in the hippocampus and cerebellum increased in a step-like manner. The accumulation of silver in the remnant was the most monotonically increasing function.

Table 2 shows the values of the silver content in the internal organs.

The silver content in a number of samples such as heart, kidney and spleen for all periods of administration was below the detection limit, which did not allow statistical processing of the results (Table S3, Supplementary Materials). Therefore, the results are not presented.

**Figure 5:**
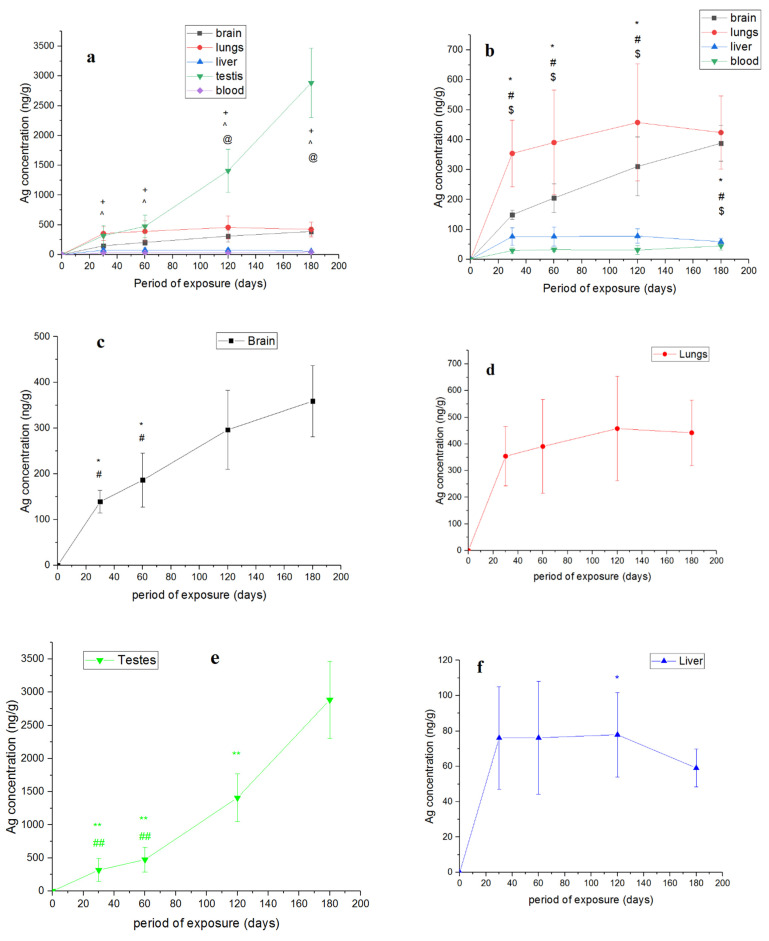

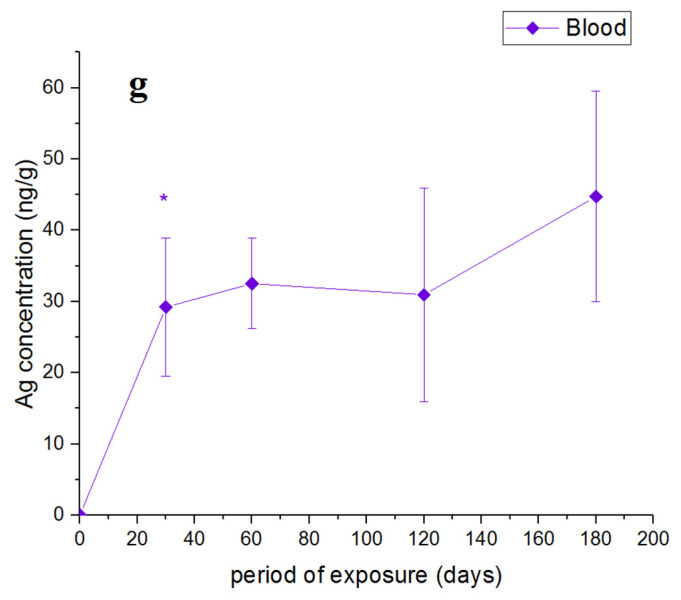
Dependence of the concentration of silver in the internal organs in the period of nanoparticle administration: (**a**) all organs (^+^ *p* < 0.05—testes vs. liver, ^^^ *p* < 0,05—testes vs. blood, and ^@^ *p* < 0.05—testes vs. brain in each group); (**b**) all organs except testes (* *p* < 0.05—brain vs. blood, ^#^ *p* < 0.05—lungs vs. liver, and ^$^ *p* < 0.05—lungs vs. blood in each group); (**c**) brain; (**d**) lungs; (**e**) testis; (**f**) liver; (**g**) blood (the points to the left denoted by * are statistically different from the 180 day point; the points to the left denoted by ^#^ are statistically different from the 120 day point; the points to the left denoted by ^$^ are statistically different from the 60 day point, * *p* < 0.05, ^#^ *p* < 0.05, ^$^ *p* < 0.05, ** *p* < 0.005, ^##^ *p* < 0.005). Number of organs per time point *n* = 6–8.

The highest absolute silver content, as can be seen, was observed in the testes (Table 2). The silver concentration in the testes also had notably high values for the periods of administration of 120 and 180 days (Figure 5a).

High concentrations of silver were also observed in the lungs and brain. The silver concentrations in the blood and liver were minimal, which may be due to the rapid washing out of silver from these organs [17] as a result of the presence of a large number of cells of the immune system and relatively fast metabolism. While in the first days of nanoparticle administration, the highest concentrations of silver are observed in the liver [25], the highest concentrations of silver in the blood are observed in the first hours of administration during acute exposure [26]. The rapid exchange between organs determines the rapid cleansing of a potential toxin. Thus, our results show that after two-month oral administration of silver nanoparticles and 1 month after its withdrawal, 80 and 75% of silver was washed out from the blood and liver, respectively, while from the brain only 5% of silver was washed out [17]. Relatively high concentrations of silver in the brain were also observed after such a long exposure to nanoparticles (Figure 5b). This may be due to the low degree of elimination from the nervous tissue [27,28].

After 120 days of administration, an extremely high accumulation of silver in the testes was observed, which may be associated with the physiological and cytological features of the blood–testicular barrier. The significant accumulation of silver in the testes is probably due to the low degree of elimination from this tissue. For example, in [27], it was found that, after 28 days of the administration of 10 and 25 nm-sized silver nanoparticles to rats and 4 months after withdrawal, a relatively low level of silver elimination from the testes was observed. The same effect was found in [28], where two types of silver nanoparticles were used (<15 nm and <20 nm). It is likely that silver nanoparticles penetrate the blood–testicular barrier, but due to its features causing a low degree of elimination, significant accumulation of silver in the testes occurs [28]. Figure 5b also shows the relatively high concentrations of silver in the lungs, exceeding the concentration of silver in the brain. The effect of silver nanoparticle accumulation in the lungs was also examined in [29], where mice were exposed to silver nanoparticles (size was around 10 nm) intraperitoneally for 21 days. This can be explained by a good blood supply to the lungs. Overall, it may be concluded that an optimal size from 1 to 100 nm provides penetration of the particles through histohematological barriers and accumulation inside tissues. We assume that nanoparticles mimic cellular organelles and proteins in the native state and are mistakenly recognized as food by the cell [30].

Previously, a significant accumulation of silver in the testes was found after 4 month of administration of silver nanoparticles plus 4 month of recovery [27] as well as 28 days of silver nanoparticle administration plus 8 weeks of recovery [28]. In this work, the effect of high accumulation of silver in the lungs was noted for the first time. The effect of the accumulation of silver in the brain has also been confirmed [17,21,22,30,31]. Previously, the kinetics of silver accumulation in different organs has not been studied for such long periods of silver nanoparticle administration (up to 180 days).

We also aimed to determine whether the curves of accumulation of silver in the organs and their regions reach saturation over the specified period. For this, the values for all periods of administration were compared using the Mann–Whitney test. The points were significantly different at *p* < 0.05. The corresponding data for the parts of the brain are shown in Figure 4b–e, and those for internal organs, in Figure 5c–f. The p values for Figure 4b–e are given in Table S4 (Supplementary Materials), and those for Figure 5c–f are presented in Table S5 (Supplementary Materials).

It can be observed that there was a tendency for the hippocampus to reach saturation starting from day 120. For the cerebellum, there was a significant saturation outcome starting from day 120. Saturation in the cortex occurred prior to reaching the 60-day point. Saturation in the remnant occurred at 120 days, as in the whole brain (Figure 5b), although the first stage of saturation had already occurred in the remnant by the 60-day point. The kinetics of accumulation in the hippocampus, cerebellum, remnant and whole brain had a similar trend.

It can be observed that saturation in the brain had already occurred by 120 days of administration, while in the lungs, it appears that saturation was achieved earlier than the considered time intervals. Saturation in the testes was not achieved in the considered periods of nanoparticle administration.

The liver showed a different tendency from that of the other organs. Saturation appeared to have occurred earlier than the considered time intervals. At 180 days, on the contrary, statistically significant excretion was observed, which is in accordance with the hypothesis of a high degree of liver self-purification due to the presence of a large number of immune system cells [17]. The kinetics of accumulation in the blood most likely has a saturation plateau, which lies below the considered values. The fact that there are several saturation plateaus (oscillations) in the kinetics of silver accumulation in different organs must also be considered.

A high level of silver accumulation in certain organs can have a toxic effect on them and negatively affect their functioning, as well as have an indirect effect on the functions of other organs and the organism as a whole. So, when mice were orally exposed to citrate-coated silver nanoparticles (10 nm) at doses of 0.25 and 1 mg/kg for 4 weeks, dose-dependent lymphopenia and an increase in triglyceride and urea levels were recorded [32]. Sprague Dawley rats were orally exposed to silver nanoparticles (8.94–33.4 nm) for 6 months at doses of 5.36 and 13.4 mg/kg. A significant decrease in testosterone levels, an increase in luteinizing hormone levels, a decrease in superoxide dismutase activity, an increase in malondialdehyde levels, and a decrease in sperm viability were found [33]. Intraperitoneal administration of silver nanoparticles (less than 100 nm) led to dose- and time-dependent damage to the tissues of the testes of rats [34]. Additionally, intraperitoneal administration of silver nanoparticles (250 nm) to Wistar rats in amounts of 30, 125, and 300 mg/kg led to dose-dependent adverse effects on sperm and seminiferous tubules: a significant decrease in the number of spermatozoa, their viability and morphology. In rats receiving the maximum dose of nanoparticles, a significant decrease in the number of spermatogonia, Sertoli and Leydig cells was also observed [35]. Thus, the accumulation of silver nanoparticles in the testes can negatively affect the ability to produce offspring. Additionally, the intake of silver nanoparticles (34.9 nm) can negatively affect the offspring due to the permeability of the placental and mammary gland barriers for them when females are exposed during pregnancy and lactation [36].

As shown in a number of studies, the accumulation of silver nanoparticles in the brain can lead to the impairment of cognitive and behavioral functions [37,38,39,40]. Thus, in a certain study [37], when orally administered to rats for 28 days, silver nanoparticles (20 nm) stabilized with bovine serum albumin at doses of 1 and 30 mg/kg showed an impartment of short-term and long-term memory. In this case, the accumulation of silver was found in the ionic form in the brain, in particular in the hippocampus, while there was no silver found in many other brain regions. Additionally, the negative effect of oral administration of silver nanoparticles (34 nm) at a dose of 2 mg/kg of body weight for 180 days on the long-term contextual memory of C57Bl/6 mice, associated with the accumulation of silver in the brain and loosening of the CA2 region of the hippocampus, was found in [30,38]. A decrease in memory and learning ability, a negative effect on social behavior and a decrease in motor functions in BALB/C mice were found after intravenous administration of silver nanoparticles [39]. The neurotoxic effect of nanoparticles appears to be due to the ability of silver nanoparticles (34 nm) to cross the blood–brain barrier [22].

The accumulation of nanoparticles in the lungs can likely have a negative effect on the respiratory system of the body; however, these phenomena after oral administration of nanoparticles are insufficiently studied and are not described in the literature due to insufficient data on the accumulation of silver nanoparticles in the lungs. However, the pulmonary toxicity of silver nanoparticles when administered by inhalation is well known and described [40,41]. For instance, after intratracheal instillation of 50 nm- or 200 nm-sized polyvinyl pyrrolidine-coated silver nanoparticles into rat organisms, reversible inflammation, DNA damage, accelerated cell proliferation and progressively increasing numbers of neutrophilic granulocytes were detected [42]. Silver accumulation was significant in homogenates of liver and other peripheral organs upon lung dose of ≥ 75 µg. Moderate pulmonary toxicity of 15 nm silver nanoparticles was found after nose-only exposition in rats. A 175-fold increased influx of neutrophils in the lungs, a doubling of cellular damage markers in the lungs, a 5-fold increase in pro-inflammatory cytokines and a 1.5-fold increase in total glutathione at 24 h after exposure were found. No effects were observed after exposure to 410 nm silver particles [43].

In addition, the exchange of nanoparticles between organs after their accumulation in the body is possible [44,45], which also implies a contribution to toxicity.

This work for the first time shows the kinetics of the accumulation of silver in different organs and their departments after long-term oral administration (up to 180 days). It demonstrates extremely high levels of silver accumulation in the testes and significant accumulation in the lungs and brain and their regions. Previously, a significant accumulation of silver in the testes and the brain was found only after the withdrawal of nanosilver [17,27,28]. The reasons for the similar affinity of silver nanoparticles to tissues should be studied further. This work also demonstrates the moments of achievement of plateau by silver accumulation kinetics.

Thus, exposure to silver nanoparticles and their accumulation in organs can be rather hazardous for those tissues and the organism as a whole. These questions require careful further research.

## 5. Conclusions

In this work, with the application of a highly sensitive and representative technique of INAA, the kinetics of silver accumulation in the internal organs and regions of the brain were obtained after prolonged (from 1 to 6 months) oral administration of silver nanoparticles at a dose of 50 µg/day/animal. Mice developed and gained weight normally during the experiment. No adverse effects were observed. An extremely high accumulation of silver in the testes was found, indicating that the long-term use of silver nanoparticles is potentially harmful to the reproductive system. A significant accumulation of silver in the lungs and brain was also found. The obtained results indicate that the main target organs during long-term administration of silver nanoparticles are the testes, lungs and brain. Additionally, it was observed that from the 180-day point, even with constant exposure to nanoparticles, silver is washed out of the liver. Relatively low silver concentrations were also observed in the blood, in which saturation appeared to have been achieved earlier. In almost all parts of the brain, a similar tendency of the kinetics of accumulation of silver to that of the whole brain was observed: saturation was achieved 120 days after beginning nanoparticle administration. A different trend was observed in the cortex, in which saturation occurred prior to the 60-day point of the administration of silver nanoparticles.

## Figures and Tables

**Table 1:** Silver masses in the different brain regions of experimental animals. Number of organs per each time point *n* = 4.

Period of Exposure, Days	Silver Mass, ng (Mean ± SD)			
	Hippocampus	Cerebellum	Cortex	Remnant
30	6.4 ± 1.5	16.3 ± 3.4	10.4 ± 3.1	28 ± 2.5
60	5.8 ± 1.5	15 ± 2.4	16.9 ± 1.5	30.3 ± 7.4
120	13.4 ± 8	27.6 ± 3	20.5 ± 6.0	42.9 ± 4.2
180	12.4 ± 2.3	8.2 ± 2.1	31.9 ± 11.3	65.9 ± 14.3

**Table 2:** Mass of silver in the internal organs and blood for different periods of nanoparticle administration. Number of organs per each time point *n* = 6–8.

Period of Exposure, Days	Silver Mass, ng (Mean ± SD)				
	Brain	Lungs	Testis	Liver	Blood
30	63.1 ± 5.3	63.7 ± 19.8	61.8 ± 33.2	105 ± 38.3	18.5 ± 5.5
60	84.7 ± 19.1	78.1 ± 34.2	87.8 ± 31.5	102.1 ± 40.9	20.1 ± 3.7
120	137.3 ± 41.2	96.1 ± 38.7	267.8 ± 66.7	113 ± 33.4	19.1 ± 9
180	166 ± 24	89 ± 19.3	519.1 ± 95.4	90.7 ± 15.1	32.2 ± 8.8

## Data Availability

Data Availability Statement: Data can be available upon request from the authors.

## Supplementary Materials

The following are available online at https://www.mdpi.com/article/10.3390/nano11123204/s1, Figure S1: Body weight of animals for different periods of exposure to nanoparticles: a–30 days, b–60 days, c–120 days, d–180 days. Table S1: Average masses of brain parts, g. Table S2: Average masses of brain parts. Table S3: Mass of silver in organs, ng. Table S4: Values of p at different time points shown in Figure 4b–e. Table S5: Values of p at different time points shown in Figure 5c–g.
