# Supplementary material for: Kinetics of Silver Accumulation in Tissues of Laboratory Mice after Long-Term Oral Administration of Silver Nanoparticles
Source: Nanomaterials (Basel). 2021 Nov 26;11(12):3204. doi: 10.3390/nano11123204 (PMC8708219; doi:10.3390/nano11123204)
Supplement: Supplementary file 1 [file nanomaterials-11-03204-s001.zip › nanomaterials-1435135-supplementary.pdf]

Supplementary Materials

Kinetics of Silver Accumulation in Tissues of Laboratory Mice after Long-term Oral Administration of Silver Nanoparticles

Anna A. Antsiferova [1,2,*], Marina Yu. Kopaeva [1], Vyacheslav N. Kochkin [1] and Pavel K. Kashkarov [1,2,3]

1 National Research Center “Kurchatov Institute”, 1, Akademika Kurchatova sq., Moscow 123182, Russia; m.kopaeva@mail.ru (M.Y.K.); Kochkin_VN@nrcki.ru (V.N.K.); kashkarov_pk@nrcki.ru (P.K.K.)

2 Moscow Institute of Physics and Technologies, 9, Institutskii Lane, Moscow Region, Dolgoprudny 141700, Russia

3 Department of Physics, Lomonosov Moscow State University, GSP-1, Leninskiye Gory, 119991 Moscow, Russia

* Correspondence: antsiferova_aa@nrcki.ru

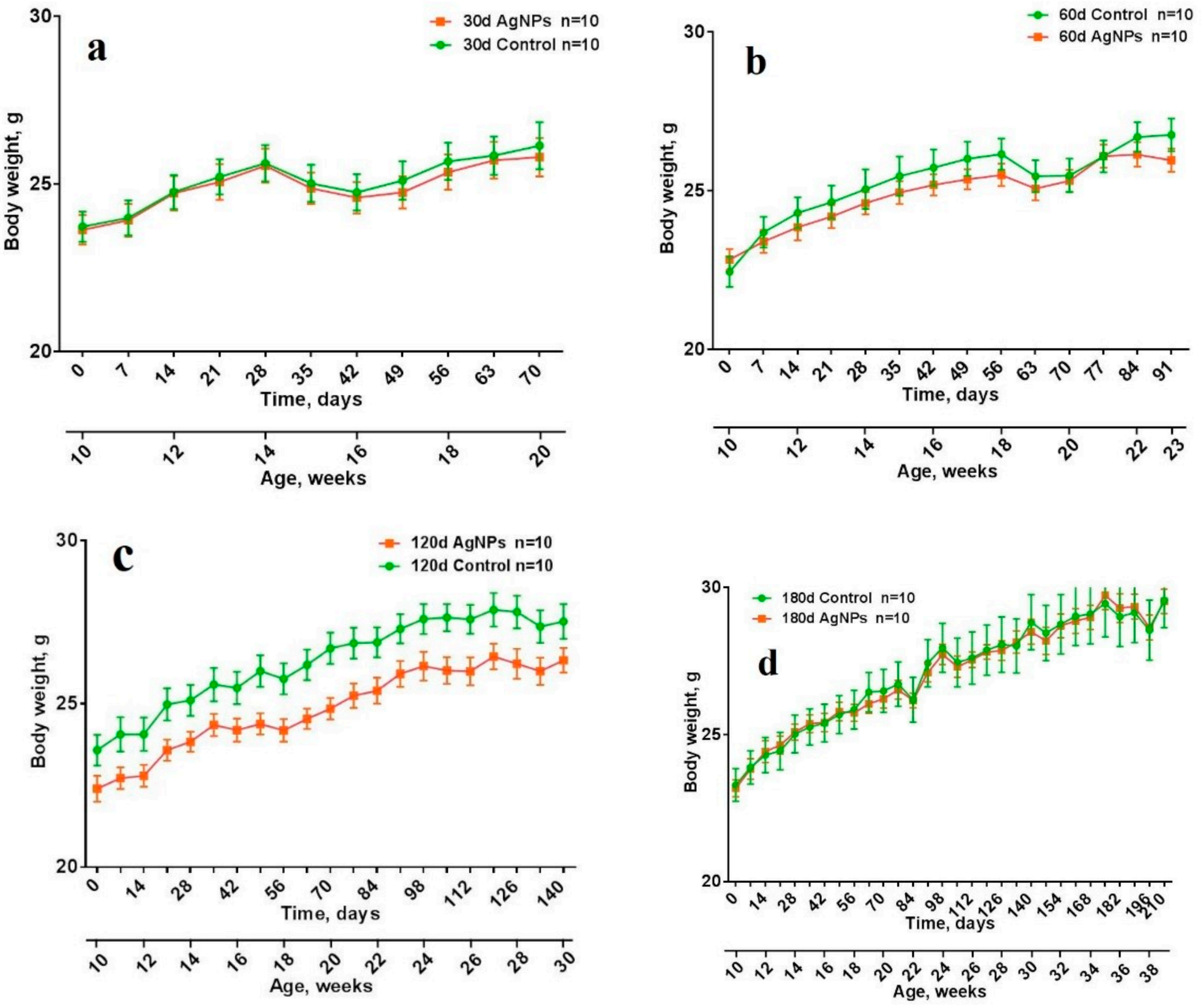

Figure S1. Body weight of animals for different periods of exposure to nanoparticles: a – 30days, b – 60 days, c – 120 days, d – 180 days.

Table S1. Average masses of brain parts, g.

Period of administration, days	Hippocampus	Cerebellum	Cortex	Remnant
30	0,054	0,136	0,155	0,225
60	0,056	0,119	0,156	0,199
120	0,053	0,113	0,133	0,201
180	0,0475	0,069	0,223	0,2605

Table S2. Average masses of internal organs, g.

Period of administration, days	Brain	Lungs	Testis	Liver	Blood
30	0,45	0,18	0,195	1,38	0,633
60	0,42	0,2	0,185	1,34	0,618
120	0,42	0,21	0,19	1,45	0,617
180	0,44	0,21	0,18	1,535	0,72

Table S3. Mass of silver in organs, ng.

N of mice	Ag mass, ng		
	kidney	heart	spleen
30d-11	<1,2	<1,4	<0,7
30d-12	<3,7	<3,2	<4,0
30d-13	<9,3	<0,3	<2,8
30d-14	<9,2	<1,7	<3,1
30d-15	<5,2	<0,4	<3,2
30d-16	23,0	<0,6	<0,8
60d-11	<7,0	<1,7	<0,6
60d-12	<4,7	<2,1	<0,6
60d-13	13,5	<2,3	<2,2
60d-14	<10	<2,9	<1,6
60d-15	27,4	<0,2	<2,7
60d-16	12,1	<1,0	<1,4
120d-11	25,7	<0,3	14,5
120d-12	<7,0	<2,9	<3,3
120d-13	<9,3	<1,4	<7,5
120d-14	12,0	<1,4	<4,2
120d-15	<6,8	<2,3	<2,3
120d-16	14,6	<3,1	<7,2
120d-17	11,1	<2,8	19,1
180d-11	<0,9	<3,8	<6,5
180d-12	12,9	<4,7	<3,0
180d-13	10,2	<2,4	<3,1
180d-14	<0,9	<1,9	<6,7
180d-15	<3,2	<2,2	<10
180d-16	14,8	<0,3	10,6
180d-17	<6,2	<1,6	<8,9
180d-18	14,7	<2,5	<6,4

Table S4. Values of p at different time points shown in Figure 4b-e.

Time intervals	p			
	Hippocampus	Cerebellum	Cortex	Remnant
30-60	0,2	0,35	0,049	0,049
30-120	0,2	0,049	0,049	0,049
30-180	0,03	0,03	0,049	0,049
60-120	0,2	0,05	0,2	0,049
60-180	0,03	0,03	0,2	0,03
120-180	0,43	0,3	0,4	0,11

Table S5. Values of p at different time points shown in Figure 5c-g.

Time interval	p				
	Brain	Lungs	Testis	Liver	Blood
30-60	0,2	0,94	0,18	0,94	0,76
30-120	0,03	0,14	0,0012	>0,9999	0,945
30-180	0,03	0,18	0,0007	0,11	0,02
60-120	0,03	0,29	0,0012	0,945	0,93
60-180	0,03	0,49	0,0007	0,08	0,11
120-180	0,1	0,95	0,0003	0,04	0,092
